# Numerical simulation of the impact of an integrated renovation project on the Maowei Sea hydrodynamic environment

**DOI:** 10.1038/s41598-021-96441-1

**Published:** 2021-08-23

**Authors:** Cui Wang, Ling Cai, Yaojian Wu, Yurong Ouyang

**Affiliations:** 1 Third Institute of Oceanography, Ministry of Natural Resource, Xiamen, 361005 China; 2 Fujian Provincial Key Laboratory of Marine Ecological Conservation and Restoration, Xiamen, 361005 China

**Keywords:** Environmental impact, Physical oceanography

## Abstract

Integrated renovation projects are important for marine ecological environment protection. Three-dimensional hydrodynamics and water quality models are developed for the Maowei Sea to assess the hydrodynamic environment base on the MIKE3 software with high resolution meshes. The results showed that the flow velocity changed minimally after the project, decreasing by approximately 0.12 m/s in the east of the Maowei Sea area and increasing by approximately 0.01 m/s in the northeast of the Shajing Port. The decrease in tidal prism (~ 2.66 × 10^6^ m^3^) was attributed to land reclamation, and accounted for just 0.86% of the pre-project level. The water exchange half-life increased by approximately 1 day, implying a slightly reduced water exchange capacity. Siltation occurred mainly in the reclamation and dredging areas, amounting to back-silting of approximately 2 cm/year. Reclamation project is the main factor causing the decrease of tidal volume and weakening the hydrodynamics in Maowei Sea. Adaptive management is necessary for such a comprehensive regulation project. According to the result, we suggest that reclamation works should strictly prohibit and dredging schemes should optimize in the subsequent regulation works.

## Introduction

The bay area is not only rich in natural resources and ecological service value, but also has excellent harbor construction conditions. It is the core area of social and economic development in coastal areas. However, due to the vulnerability of ecological environment and the rapid growth of human activities, the hydrodynamic environment of the bay has undergone a series of changes, and the Marine resources and ecological environment have been damaged to different degrees^1^, which seriously affect the sustainable development of the Marine environment^2,3^. Therefore, it has become one of the effective scientific research strategies for the coastal countries and regions to study deeply the problems existing in the bay and carry out the comprehensive regulation of the bay. According to the 2010 Guidelines on the Improvement, Restoration, and Protection of Sea Areas, Islands, and Coastal Zones prepared by the State Ocean Administration, improving the coastal landscape ecology and promoting sustainable development in social usage of the sea through renovation and restoration projects are essential. The hydrodynamic conditions are the important environmental factors in marine area^4^ and directly relate to the ecological environment and associated with economic development. Therefore, it is of great significance to study the influence of the integrated renovation project on hydrodynamics environment of the bay.

The Maowei Sea in the north Qinzhou Bay in Guangxi Province, China, is a typical semi-closed natural bay, characterized by a wide interior and narrow mouth. It covers approximately 135 km^2^, with an almost 120 km-long coastline and an average water depth less than 10 m^5^. A huge tidal prism maintains a tidal channel, which can cause strong tidal current and produce a 10^4^-ton berth in the Qinzhou Port. Owing to freshwater discharged from the Qinjiang River and Maoling River, the Maowei Sea area contains plants typical of an estuarine wetland ecosystem such as mangroves and salt marshes. As a major germplasm resources reserve, especially for the Jinjiang oyster, the area is known as "the hometown of large oysters in China" and hosts abundant large oysters, green crabs, prawns, and groupers^6^. However, because of rapid industrialization, increasing population, and excessive mariculture in recent years, the Maowei Sea area experienced mounting environmental challenges such as a shrinking area, weakened hydrodynamics, eutrophication, and heavy metal pollution^7,8^. These problems threatened the sustainable development of common activities such as fisheries.

**Figure 1:**
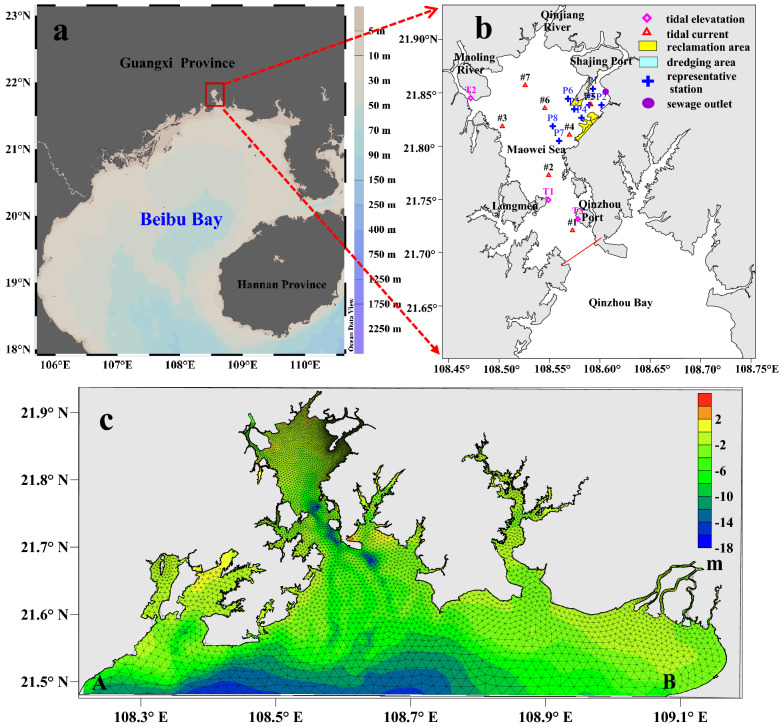
Map showing the location **(a)**, the investigated stations **(b)** and the triangular mesh **(c)** in the Maowei Sea. These figures were plotted using the Ocean Data View (Schlitzer, R., https://odv.awi.de, 2018) **(a)** and Surfer10 Software (https://www.goldensoftware.com/products/surfer) **(b,c)**.

The *Integrated Renovation Plan of the Maowei Sea* was issued by the Qinzhou Municipal Government in 2010. Several integrated renovation and restoration projects were conducted in the area in the past decade, including dredging and reclamation of tidal flats, beach restoration, bank revetments, and mangrove habitat protection. Dredging was performed in an area of the Maowei Sea covering almost 14 km^2^, producing a dredging volume of approximately 15.91 × 10^6^ m^3^. The dredged soils served in the construction of an area covering 4.42 km^2^, producing an extension of the shoreline by approximately 4.2 km (Fig. 1). These projects focused on improving the water quality of the Maowei Sea, preserving its unique ecological and species diversity, building a livable and commercial bay city, and enhancing the value of the marine resources^9^.

At present, many research results have been published about the hydrodynamic environmental variation after the projects have been done because all kinds of marine projects have been implemented^10,11^. In the coastal and estuarine waters, the tidal current is the main dynamical factors^12^. Numerical simulation of hydrodynamics is widely applied in studies of coastal and estuarine problem^13,14^. For example, Shen et al.^15^ have predicted the influence of reclamation and showed that the reclamation had a great influence in the Pearl River Estuary base on the FVCOM Module. Cao et al.^2^ have predicted the impacts of the reclamation project by using MIKE3 model. Zhang et al.^6^ have analyzed the change characteristics of water exchange and showed the tidal prism increased after dredging in Maowei Sea. Ren et al.^16^ studied an erosion-siltation numerical simulation after ecological restoration engineering in Maowei Sea. Yang et al.^17^ discussed the influence of shoreline change on dynamics and water exchange in Maowei Sea. The results showed the tidal range and flow velocity decreased, the water exchange capacity were weakened. In addition, some indicators have been utilized in hydrodynamic environmental impact assessments of coastal projects, e.g., tidal elevation^18^, current speed^19^, tidal prism^20^, water exchange capacity^21^, sediment deposits and water quality^22,23^ (Table 1). Although almost every coastal project in Maowei Sea have been individually simulated by numerical model to analyze the change information, this research studies the accumulated impact of the large-scale integrated renovation project of Maowei Sea in the past 10 years as a whole. In this study, the MIKE3 software is employed to construct three-dimensional (3D) hydrodynamic and water quality models with the use of high-resolution triangular meshes (approximately 30 m) fitting the shoreline. The aims of our study are to explore the impacts of an integrated project on the hydrodynamic, tidal prism, water exchange capacity, and the erosion and deposition characteristics of the Maowei Sea environment. The results of this study can expect to provide an assessment of the effects of a remediation project and provide a basis for the implementation and optimization of subsequent ecological restoration projects in the Maowei Sea.

**Table 1:** Comparison of the numerical models used in the Maowei Sea.

Researchers	Model	Research content
Zhang et al.^6^	2D hydrodynamic and water quality model with MIKE 21	The change characteristics of water exchange and the tidal prism increased after dredging of Qinzhou Bay
Ren et al.^16^	2D hydrodynamic and erosion-siltation model with MIKE 21	The change of current field and sediment transport caused by ecological restoration engineering of Maowei sea
Yang et al.^17^	2D hydrodynamic and water quality model with MIKE 21	The influence of shoreline changes on dynamics and water exchange of Maowei sea
Dong et al.^18^	2D hydrodynamic model	The changes of current speed and water volume of Qinzhou Bay caused by reclamation
Jiang et al.^19^	A hydrodynamic model with FVCOM	The characteristics of current and water exchange in Qinzhou Bay
Guo et al.^20^	2D hydrodynamic and water quality model	The change of tidal prism and water exchange by reclamation of Qinzhou bay mouth
Chen et al.^21^	3D hydrodynamic and water quality model with POM	Study the water exchange capacity and half-life time in the Qinzhou Bay
Wang et al.^22^	3D hydrodynamic and particle tracking model	Analysis of seasonal characteristics of water exchange in Beibu Gulf
He S.^23^	2D hydrodynamic and water quality model with MIKE 21	Study on water quality and environmental capacity of Maowei Sea
This study	3D hydrodynamic and water quality model with MIKE 3	Study the impact of hydrodynamic environment (current speed, tidal prism, water exchange capacity, sediment erosion and deposition) by an integrated renovation project in Maowei Sea

## Materials and methods

### Hydrodynamic model construction

A numerical hydrodynamic model was established using the MIKE3 software developed by the Danish Hydrodynamics Laboratory. The mathematical foundation of MIKE3 is the Reynolds-averaged Navier–Stokes equations and the mass conservation equation, which use Alternating Direction Implicit (ADI) technique to integrate equations for mass and momentum conservation in the space–time domain^24^. The model can fit the shoreline infinitely by using triangular mesh. This software is widely utilized for simulating the water quality and sediment dynamics in estuarine, coastal, and marine environments because of its advanced pre- and post-treatment capabilities and user-friendly interface^25,26^. In the model, the study area, covering the Maowei Sea and Qinzhou Bay (108°12′–109°11′ E and 21°27′–21°56′ N) involves a computing range of 125 km × 57 km. This area was divided into 15,456 elements and 28,048 mesh nodes using a triangular mesh, and the minimum resolution was 30 m. The area was also partitioned into four layers through a vertical sigma coordinate system, and the model time step was set to 10 s. The duration time of the model is about one year. Two different grids to analyze the environmental impact before and after the construction of the project were used in this study. The difference between the two grids mainly reflects the different coastline of the project area and the different water depth conditions of the dredging area. The open boundary located in the southern simulated area, was forced by the tidal elevation calculated using the MIKE global tide module. The predicted tide elevation was interpolated by the difference between point A and point B under given latitude and longitude coordinates. The monthly average flow was taken as the boundary value of the Maoling and Qinjiang rivers upstream. A quadratic bottom drag was used for the bottom stress with the friction coefficient of 0.01. The model performance was evaluated by the root mean square error (RMSE).

**Figure 2:**
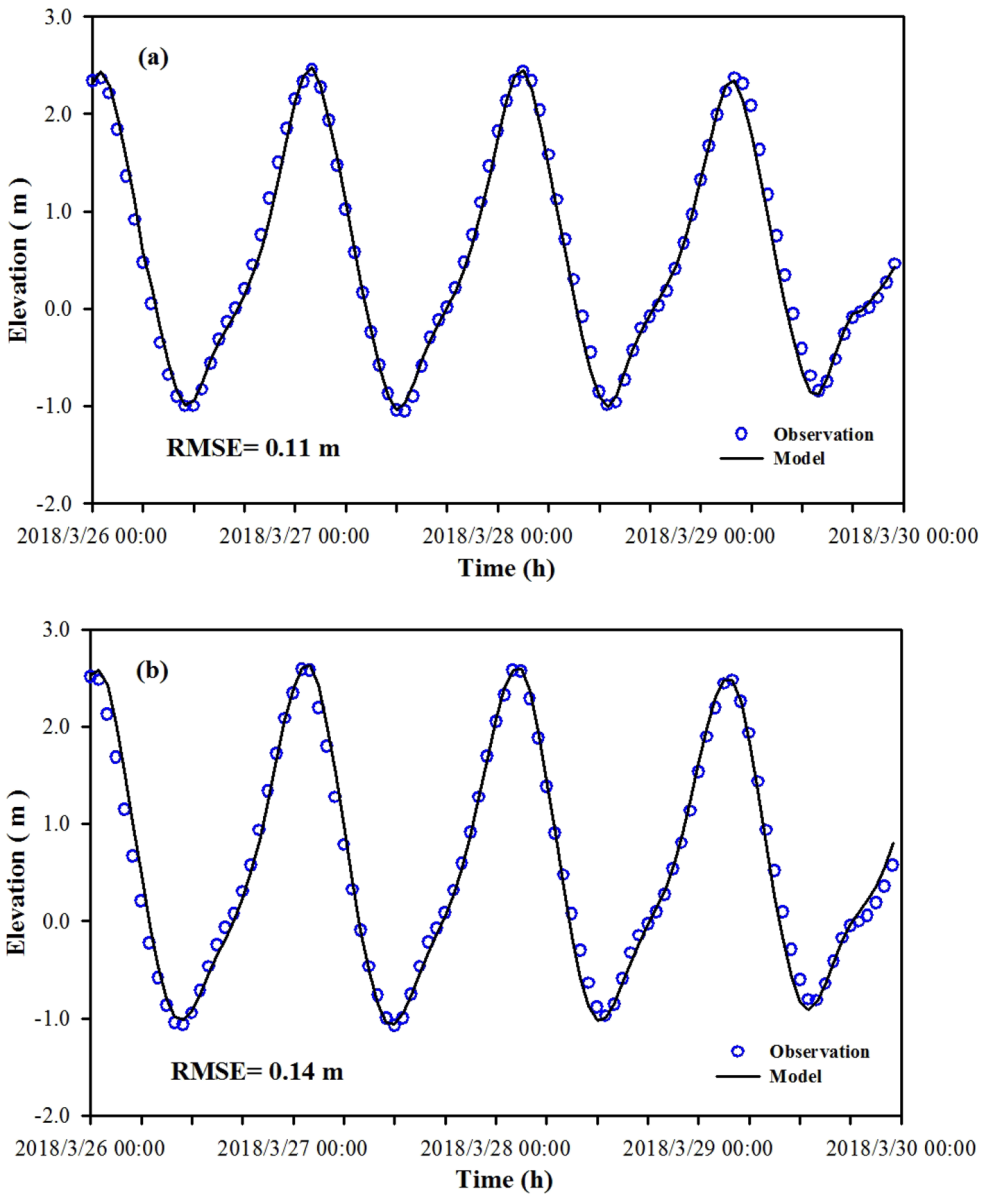
The variations of water elevation between observation and model with time (**(a)** is for T1 station and **(b)** is for T3 station). These figures were prepared with SigmaPlot Version 10.0.

**Figure 3:**
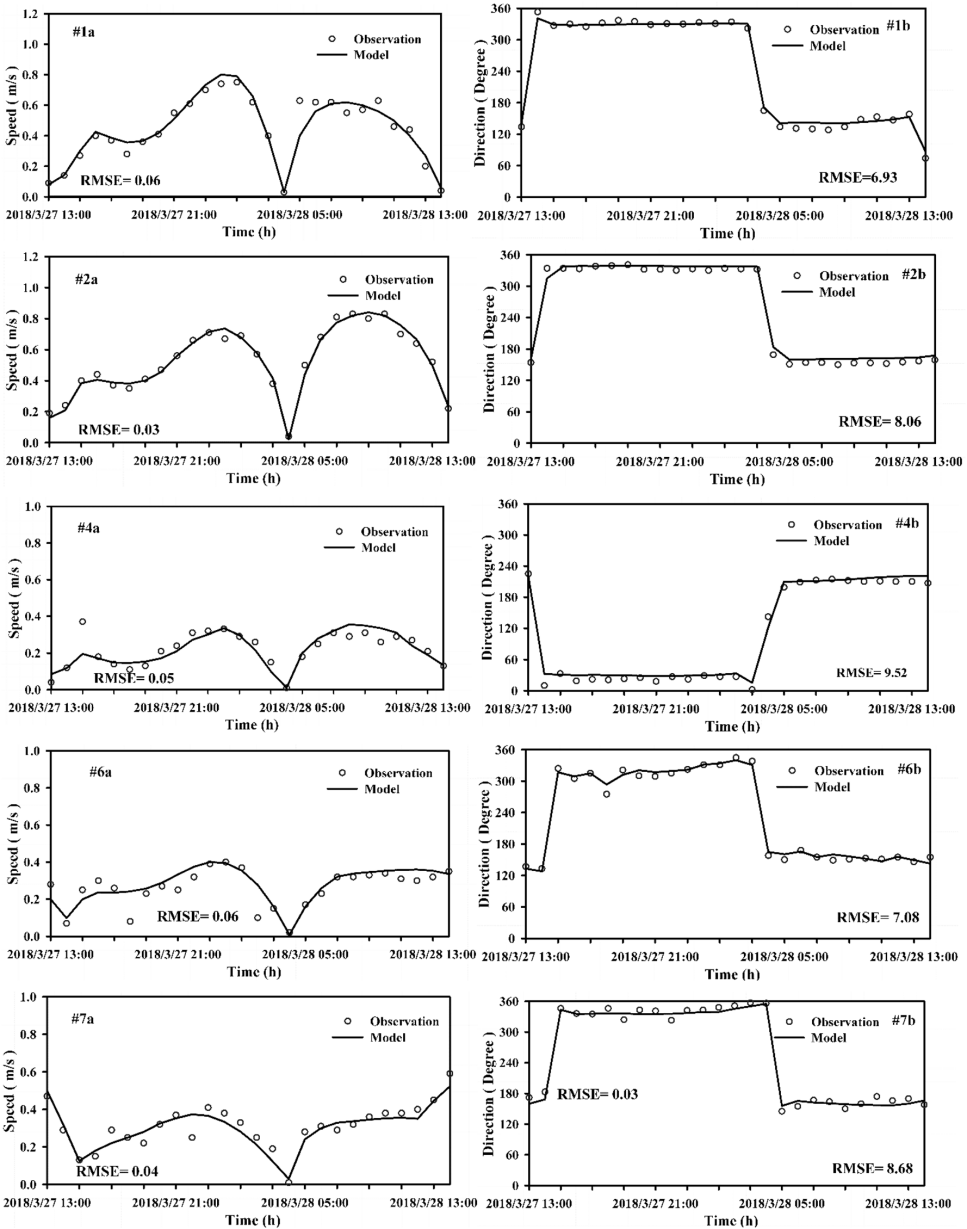
The variations of speed and direction of tidal currents in observation and model with time. These figures were prepared with SigmaPlot Version 10.0.

The 2018 hydrological monitoring results from three tidal elevations and seven tidal current stations of the Qinzhou Oceanic Monitoring System were employed for validating the Maowei Sea model (Fig. 1). The tidal elevation and tidal current validation results are shown in Figs. 2 and 3, respectively. Comparing the measured data and simulated of tidal elevation generated RMSE of approximately 0.11 m and 0.12 m. The Maximum RMSE of the current and direction was 0.06 m and 9.52°, respectively. Overall, the measured field values are consistent with the simulation results. For example, the calculated peak values and the flow velocity phases at different points generally agree with the measured results. The flow calculation results of the rising and ebbing of tides also display consistency with the measured values, although some current reversals were observed. This validation implies that the mathematical model adequately simulates flow currents in nature, thus providing a hydrodynamic drive for water quality model research.

### Tidal prism determination

The tidal prism is an important index for assessing the water exchange capacity and self-purification capacity in a marine bay^27^. It is usually calculated using the following equation:

1 $$P = \frac{1}{2}(S_{1} + S_{2} )(H_{1} - H_{2} )$$

where *P* is the tidal prism, *S*_*1*_ and *S*_*2*_ represent the water area at high and low tides, respectively, while *H*_*1*_ and *H*_*2*_ denote the high and low tide elevations, respectively.

To obtain accurate results, the tidal prism concept was applied to each grid of the model, thereby revising the tidal prism formula as follows:

2 $$p = \sum\limits_{i = 1}^{n} {S_{i} (H_{1i} - H_{2i} )}$$

where S_*i*_, *H*_*1i*_, and *H*_*2i*_ are the grid area and the water elevation of the *i*th grid at high and low tides, respectively, *n* is the number of grids in the studied sea area, and *P* is the tidal prism.

### Water quality model construction

A 3D water quality model of the Maowei Sea was constructed based on the hydrodynamic model, and the model is represented by the following basic equation:

3 $$\frac{{\partial {\text{C}}}}{\partial t} + \frac{\partial }{{\partial {\text{x}}}}(uc) + \frac{\partial }{{\partial {\text{y}}}}(vc) + \frac{\partial }{{\partial {\text{z}}}}(wc) = \frac{\partial }{\partial x}(D_{x} \frac{\partial C}{{\partial x}}) + \frac{\partial }{\partial y}(D_{y} \frac{\partial C}{{\partial y}}) + \frac{\partial }{\partial z}(D_{z} \frac{\partial C}{{\partial z}}) + Q_{s}$$

where *C* is the concentration of a dissolved conservative substance, $u, v$ and $w$ are velocity components, *D*_*x*_, *D*_*y*_, and *D*_*z*_ are the diffusion coefficients in the x-, y-, and z- directions, respectively, *Q*_*s*_ is a source or sink term; and t represents time.

### Water exchange capacity analysis

The water exchange capacity is a vital indicator for evaluating the capacity and quality of a marine bay^21^. Therefore, it was employed as an important basis for understanding the water exchange capacity of the Maowei Sea. Luff and Pohlmann^28^ introduced the concept of half-life, representing the time required for the concentration of a conservation substance to reduce to half of its initial value, and this commonly exhibits a negative correlation with the water exchange capacity. The dynamic conditions of different areas differ greatly due to the wide water area of Maowei Sea. There is a certain deviation in using the half-life time when the average concentration reduces to half its initial value. Therefore, in this study, the water quality model^17,29^ was adopted to calculate the half-life as the time when the total load decrease to half of the initial value. The total load was calculated by the equation as follows:

4 $$Q_{t} = \sum\limits_{i = 1}^{n} {(A_{i} \cdot H_{i} \cdot C_{i} } )$$

where $Q_{t}$ is the total load, and* A*_*i*_, *H*_*i*_, and *C*_*i*_ represent the area, water depth, and concentration associated with the *i*th grid, respectively.

### Siltation measurement

Owing to the water depth and flow field changes after implementation of the integrated renovation project in the Maowei Sea, the sediment-carrying capacity also changed. The associated impact on sediment suspension and deposition consequently altered the seabed erosion and deposition intensity. The Maowei Sea area represents a silty bay dominated by suspended sediment transport. Considering the flow fields before and after the project obtained from the hydrodynamic model, the formula proposed by^30^ was adopted to calculate the deposition intensity as follows:

5 $$L = (1 + \psi )\frac{{K_{1} \omega M_{1} t}}{{\gamma_{0} }}\left[ {1 - \frac{{V_{2} }}{{V_{1} }}\left( {\frac{{d_{1} }}{{d_{2} }}} \right)^{2} } \right]$$

where $L$ is the thickness of the suspended load deposited in the engineered area in time $t$, while $V_{1}$, $d_{1}$ and $V_{2}$, $d_{2}$ are the flow velocity and water depth before and after the project, respectively, $M_{1}$ is the average sediment concentration in the shoal water under a combined wave and tidal current action, $\omega$ is the sedimentation rate ($\omega$ of marine silt is attributed to the rate for flocculation of 0.0004–0.0005 m/s; while the $\omega$ of other silt is associated with a particle); $\gamma_{0}$ is the dry density of the deposit, given as $\gamma_{0} = 1750d_{50}^{0.138}$($d_{50}^{{}}$ indicates the median size of sediment), $\psi$ is the bed load deposit proportion in the suspended sediments, $K_{1}$ is the cross-flow sedimentation coefficient, with $K_{1} = 0.35$, and $t$ is the time.

## Results

### Effect on flow velocity

**Figure 4:**
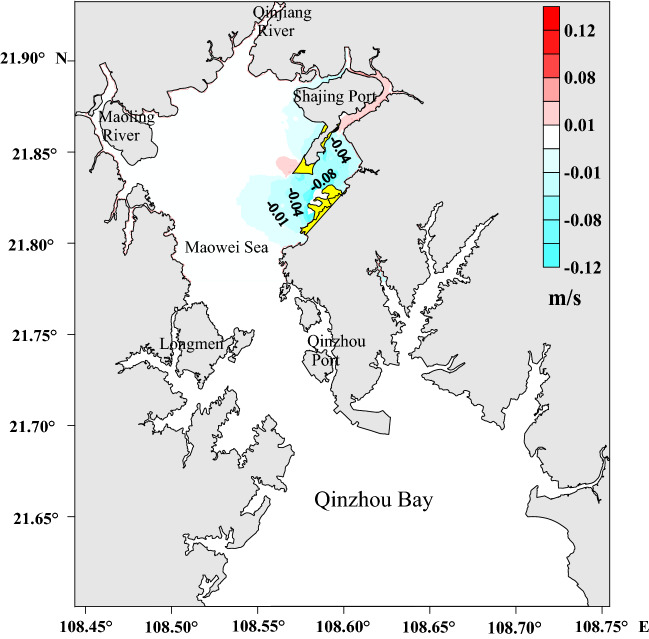
The variations of average velocity in Maowei Sea after the project (a positive value indicates an increase while a negative value means a decrease). The figure was drawn with Surfer10 Software (https://www.goldensoftware.com/products/surfer).

The average flow velocities measured before and after the project (Fig. 4) reveal the changes in the east of the Maowei Sea area after the project. The velocity around the reclamation sites decreased by ~ 0.12 m/s, while those around the dredging sites decreased by approximate 0.06 m/s. However, in the dredging area northeast of the Shajing Port, the velocity increased by approximately 0.01 m/s.

In order to evaluate the influence on hydrodynamics after the project, eight representative point (Fig. 1) were chosen for comparison of characteristic velocity, and the changes of velocity at the comparison points are shown in Table 2. The velocity comparison points were mainly distributed in the comprehensive reclamation area and dredging area of the Maowei Sea. The velocity at station P1, located in the east of Shajing Island, increased by about 0.04 m/s. While the velocity at station P2 decreased about 0.06 m/s. Note that, the velocities at stations P3, P4 and P5 near the reclamation project on the eastern of Shajing Island showed an obvious change, up to about 0.13 m/s. The velocity of P6 in the west of Shajing Island has a slight increase of about 0.03 m/s, indicating that the engineering construction has little impact on the west of Shajing Island. The velocities of P7 and P8 located outside the project changed slightly. Therefore, the velocity changes mainly occurred at reclamation and dredging sites, while other sea areas were essentially unaffected.

**Table 2:** Comparison of velocity at the representative station before and after the project.

Station	High tide			Ebb tide		
	Before	After	Variation	Before	After	Variation
P1	0.25	0.28	0.03	0.28	0.32	0.04
P2	0.09	0.04	−0.05	0.10	0.04	−0.06
P3	0.25	0.13	−0.12	0.37	0.24	−0.13
P4	0.33	0.24	−0.09	0.45	0.33	−0.12
P5	0.28	0.17	−0.11	0.31	0.23	−0.08
P6	0.32	0.37	0.05	0.37	0.40	0.03
P7	0.38	0.36	−0.02	0.41	0.38	−0.03
P8	0.41	0.40	−0.01	0.50	0.49	−0.01

### Tidal prism variations

To understand the influence of the integrated renovation project in the Maowei Sea area on the tidal volume, a section (Fig. 1) at the mouth of the Maowei Bay was used to calculate the tidal prism before and after the project. The calculations produced average tidal prism values of 3.10 × 10^8^ and 3.07 × 10^8^ m^3^ before and after the project, respectively. The dredged area of 14 km^2^ and approximately 15.91 × 10^6^ m^3^ of dredging volume involved in the project liberated nearly 16 × 10^6^ m^3^ of space, thereby enhancing the ability of the Maowei Sea to accommodate more inflowing water and elevate the tidal prism of the bay. However, beside dredging works, the project involved the reclamation of almost 4.42 km^2^ of land along the east coastline. Therefore, because this mostly occurred in the intertidal flats, the sea area reduced, thereby decreasing the tidal prism of the bay. Consequently, the combined action of dredging and reclamation decreased the tidal prism of the Maowei Sea area by almost 2.66 × 10^6^ m^3^ after the project. Nevertheless, the decrease in the tidal prism accounts for just 0.86% of the pre-project level.

### Water exchange capacity changes

**Figure 5:**
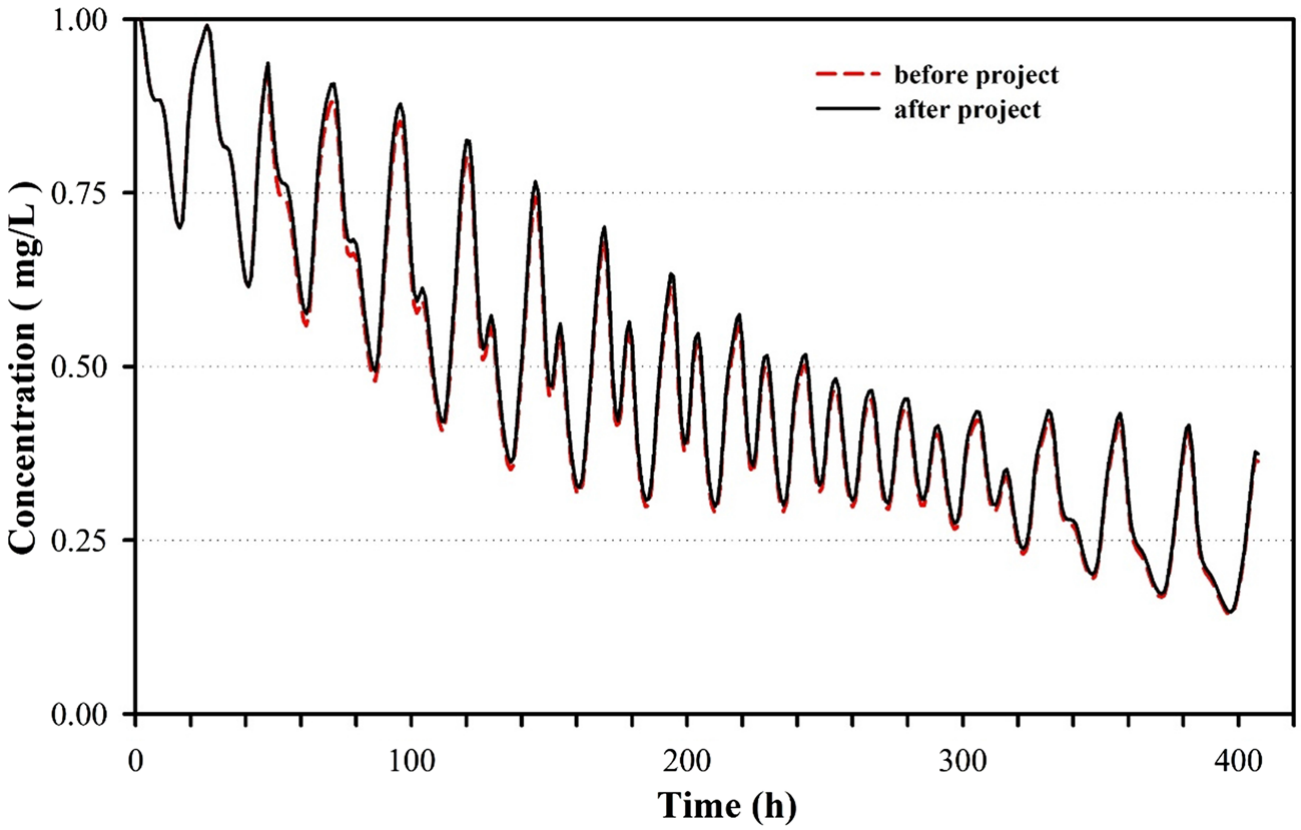
The variations of concentration of conservative substance with time in the water before and after the project. The figure was drawn with SigmaPlot Version 10.0.

The half-life was calculated by assuming a concentration of 1 mg/L for a conservative element in water of the Maowei Bay (the section is shown in Fig. 1), while the concentration in water outside the bay was considered as 0 mg/L. Continuous exchange occurred between water in and outside the bay because of the rise and fall of tides. The time for the average concentration of the conservative element in water of the bay to decrease from 1 to 0.5 mg/L was taken as the half-life. The variations in the concentration of the conservative substance in the water before and after the project are displayed in Fig. 5.

According to the water exchange modeling, the concentration in water of the bay before the project failed to exceed 0.5 mg/L after 221 h, indicating a half-life of 221 h (~ 9 day). After the project, the concentration reached 0.5 mg/L in 244 h, suggesting a half-life of 244 h (~ 10 day). These results reveal that the half-life increased by 23 h after the project. This decrease is attributed mainly to a lower water exchange capacity caused by the smaller water area and the higher water depth after the reclamation and dredging.

**Figure 6:**
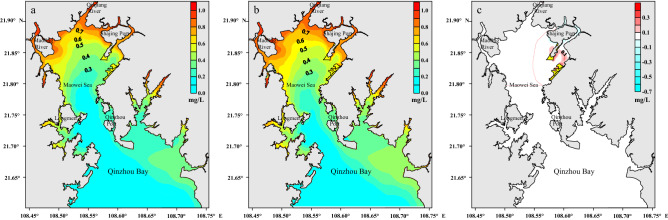
Distribution of pollutants in the Maowei Sea area at the residence half-life time before the project **(a)**, after the project **(b)** and the variation of the pollutant concentration after project **(c)**. These figures were drawn with Surfer10 Software (https://www.goldensoftware.com/products/surfer).

The spatial variation in the concentration of the conservative element in the Maowei Sea at half-life is displayed in Fig. 6. Owing to the distance from the entrance of the Maowei Bay, the northeast and northwest parts are characterized by low water exchange values. The concentration before the project in the southwest of the Shajing Port was approximately 0.6 mg/L, while in the east of the Port, the concentration varied between 0.6 and 0.7 mg/L. Therefore, except for the project area, the concentrations after the project in other areas are consistent with those before the project.

The variation of concentration at eight representative stations before and after the project is listed in Table 3. The concentration of pollutant at station P2 at the half-life time increased about 0.22 mg/L, while the concentration at other stations increased 0.02–0.12 mg/L. Overall, the concentration change area is mainly concentrated in the project scope, and the concentration has increased after the project.

**Table 3:** Comparison of concentration at the representative station before and after project.

Station	pollutant concentration (mg/L) at the half-life time			COD concentration (mg/L) near Industrial Park		
	Before	After	Variation	Before	After	Variation
P1	0.59	0.71	0.12	1.23	1.00	−0.23
P2	0.48	0.69	0.22	1.07	1.37	0.29
P3	0.39	0.43	0.04	0.36	0.22	−0.14
P4	0.39	0.42	0.03	0.13	0.12	0.00
P5	0.39	0.43	0.04	0.11	0.04	−0.06
P6	0.41	0.45	0.04	0.06	0.03	−0.03
P7	0.27	0.29	0.02	0.01	0.01	0.00
P8	0.27	0.29	0.02	0.04	0.03	−0.01

### Pollutant advection–diffusion capacity variation

**Figure 7:**
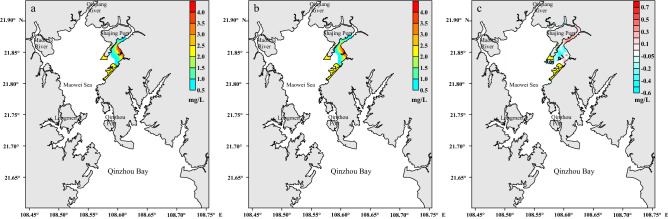
Distributions of COD concentration in the Industrial Park before the project **(a)**, after the project **(b)** and its variation after project **(c)**. These figures were drawn with Surfer10 Software (https://www.goldensoftware.com/products/surfer).

The characteristics of the advection–diffusion process are studied by the concentration tracer method. To assess the advection and diffusion of pollutants before and after the project, a sewage outlet associated with the integrated renovation project is selected for analyzing the convective diffusion of pollutants. The east sewage outlet of the Qinzhou Port Industrial Park^23^ in the northeast of the Maowei Sea area (Fig. 1) involves a flow of 2.37 × 10^8^ m^3^/year, with the chemical oxygen demand (COD) concentration of 4.0 mg/L^22^. The diffusion of pollutants through the sewage outlet of the Industrial Park before and after the project is simulated based on the water quality model, and the results are shown in Fig. 7. COD is regarded as a dissolved conservative substance without considering degradation in the model calculation.

The COD of the sewage in the outlet before the project exceeded the Class II seawater quality standard (COD concentration > 3 mg/L) and covered an area of approximately 0.83 km^2^, while the COD exceeding the Class I seawater quality standard (COD concentration > 2 mg/L) values covered almost 1.75 km^2^. After the project, the area with COD higher than that of the Class II seawater quality standard decreased to approximately 0.73 km^2^, while the area with COD exceeding the Class I seawater quality standard value reduced to approximately 1.67 km^2^. After the project, the concentration of COD at station P2 increased about 0.29 mg/L, while the concentration at other stations decreased 0–0.23 mg/L. The COD concentration in most areas of the eastern part of Shajing Island has been reduced, and the COD concentration in the northeast side of the reclamation area increased slightly. Overall, this indicated that the project could promote the transport and diffusion of pollutants out of these areas, and thus, helping in pollution control and improving the quality of the environment.

### Sediment erosion and deposition

Maowei Sea is a continental fluvial depositional landform. Due to the sediment deposited near the mouth of the Qinjiang River and Maoling River, it has been continuously pushed to the sea, forming extensive sandy and silt intertidal shoals, tidal trenches, estuarine bars, and deep tidal trenches, which is roughly distributed in a north–south direction^31^. The coastal area of Maowei Sea is mainly muddy coast, with different characteristics of bedrock, sandy shore and sandy mud shore. The main sedimentation occurred in the inner Bay of Maowei Sea, and the sedimentation rate was about 0.17 cm/year^32^.

**Figure 8:**
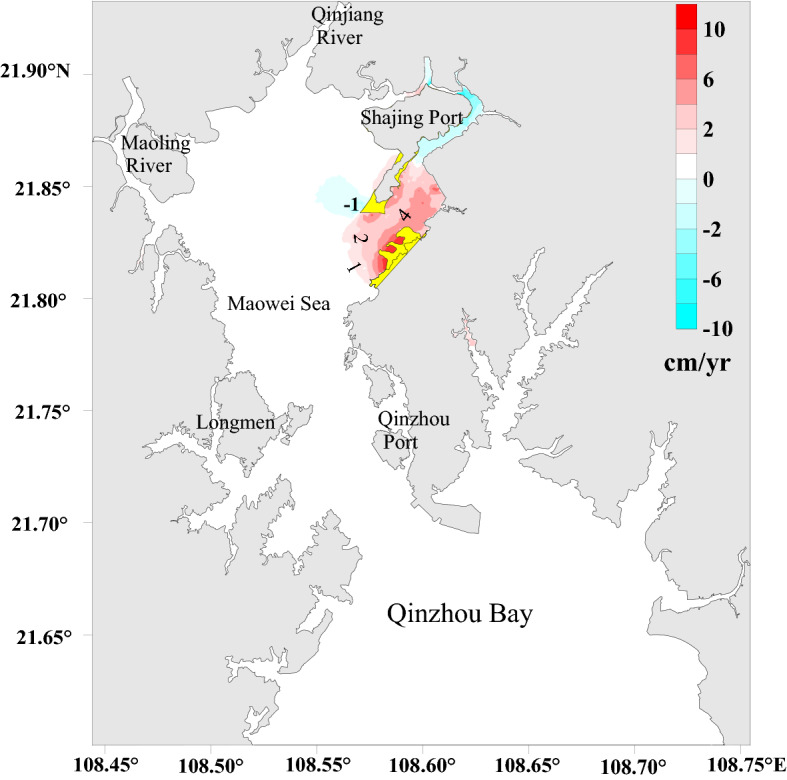
The variations of erosion and deposition in sediment after the project. The figure was plotted with Surfer10 Software (https://www.goldensoftware.com/products/surfer).

According to the sediment erosion and deposition values distribution after the project (Fig. 8), the east Maowei Sea is characterized by moderate siltation intensity and volume. The maximum siltation of 10 cm/year occurs around the reclamation area in the east Maowei Sea, especially in the concave area of the zigzag shoreline linked to the reclamation work. The back-silting volume near the dredging area is approximately 2 cm/year. The higher flow rate caused a sediment scour of approximately 1 cm/year in the northeast of the Shajing Port. The change in the filling shoreline created a small siltation area in the southwest of the port, with a silting volume of almost 1 cm/year. In general, the lower post-project flow rates promoted back-silting in most areas, although the average volume is limited to 2 cm/year. Siltation changed mostly in the reclaimed and dredged areas, with the other areas barely affected. The sediment back-silting caused by the integrated renovation project was expected to last approximately 5 years before re-establishment of the natural state of erosion and deposition in the area.

## Discussion

In this study, three-dimensional hydrodynamics and water quality models are developed for the Maowei Sea to assess the hydrodynamic environment base on the MIKE3 software with high resolution meshes. The observed tidal elevation and current measured are used for the hydrodynamic model. The RMSE for tidal elevation at Longmen station (T1) and Qinzhou station (T3) are 0.11 m and 0.14 m, respectively. The RMSE for current velocity in the stations (# 1, # 2, # 4, # 6, # 7) are ranged from 0.03 to 0.06 m, while the RMSE for current direction ranged from 6.93° to 9.52°. Although the errors are not negligible for the tidal elevation, velocity and direction, the errors are accepted within the allowable range. Maowei Sea is a semi-enclosed bay with complicated bathymetry and many islands, the input coastline and terrain for simulation may lead to some errors that may impact the simulation results. Through the verification and calibration of the model, the bottom friction coefficient is determined to be 0.01 m. This model can provide a hydrodynamic field for further study of hydrodynamic processes.

This study can well analyze the variations of the hydrodynamic environment caused by the remediation engineering, which can be a useful tool to manage integrated renovation project construction. This evaluation of the changes in Maowei Sea has been limited to the tidal current, tidal prism, and water exchange capacity. the sediment deposition was calculated with the Eq. (5) caused by the flow velocity variations. However, the impact assessment of environmental changes caused by construction is complex and variable, and it should include more factors, such as water quality, ecosystem parameters. In addition, it is necessary to build sediment transport model and biological model in the further research in Maowei Sea.

The objective of the comprehensive regulation project of Maowei Sea is to increase the tidal prism, improve the ecological environment and form a certain construction land, which has good economic, environmental and social benefits. At the present stage, the comprehensive control project of Maowei Sea has completed the dredging project with an area of 14 km^2^, which increases the three-dimensional space of nearly 16 million m^3^ in the Sea, increasing the ability of Maowei Sea to accept external sea water and the tidal capacity. On the other hand, the area of land reclaimed from the sea is about 4.42 km^2^, most of which is concentrated in the intertidal zone, resulting in the reduction of sea area. The numerical simulation results show that the implementation of the project will lead to a slight decrease in tide carrying capacity, a weakening of water exchange capacity, and sediment deposition in some areas, suggesting the hydrodynamic conditions of the bay is related to the marine area^4^. Reclamation is the main factor affecting the decrease of tidal capacity in the Maowei Sea. In order to restore the tidal capacity of the Maowei Sea and enhance the water exchange capacity, it is suggested that no new reclamation projects should be allowed and the increase of the sea area with tidal capacity should be given priority in the subsequent regulation projects of the Maowei Sea. Secondly, strengthen the land-sea overall planning, optimize the dredging scheme, restore the sea water dynamic conditions, and ensure the smooth bay waterway, with a view to achieving the regulation goals as soon as possible.

## Conclusions

Using mathematical models, the effects of an integrated renovation project on the Maowei Sea were identified by analyzing the hydrodynamic environment. According to the hydrodynamic indexes simulated before and after the project, the hydrodynamic conditions decreased significantly in the east Maowei Sea because of land reclamation and desilting. During this period, the flow velocity in the east Maowei Sea decreased by approximately 0.12 m/s, whereas in the northeast of the Shajing Port, the flow velocity increased by almost 0.01 m/s. The average tidal prism of the Maowei Sea of 3.07 × 10^8^ m^3^ after the project was 2.66 × 10^6^ m^3^ less than that before the project, accounting for a difference of just 0.86%. The half-life increased from 221 h before the project to 244 h after the project, implying that the water exchange capacity slightly decreased. Siltation was dominant in the east of the Maowei Sea, characterized by an average value of approximately 2 cm/year. The results of this study provide a basis and reference for subsequent ecological restoration projects and for the sustainable development of the Maowei Sea resources and environment.

Adaptive management capable of continuously identifying and solving problems through timely adjustment of the regulation and restoration strategies are recommended during phased implementation of integrated renovation. The results showed that the reclamation project is the main factor causing the decrease of tidal volume and the weakening of hydrodynamics in Maowei Sea. In the next integrated renovation project, we suggest that reclamation works should strictly be prohibited while dredging schemes should be utilized. Restoration project should implement to increase the tidal capacity and water exchange capacity as far as possible, to protect the Marine environment and restore the marine ecology of Maowei Sea.
